# Expectations of general practitioners on a practice based research network in Germany- a qualitative study within the Bavarian Research Practice Network (BayFoNet)

**DOI:** 10.1186/s12875-023-02239-7

**Published:** 2024-01-02

**Authors:** Linda Sanftenberg, Julia Stofella, Katharina Mayr, Armin Nassehi, Annette Härdtlein, Stefanie Stark, Thomas Kühlein, Peter Konstantin Kurotschka, Ildikò Gágyor, Stefanie Eck, Antonius Schneider, Melanie Bößenecker, Marco Roos, Tobias Dreischulte, Jochen Gensichen, for the BayFoNet study group, Andrea Baumgärtel, Isabell Endrich, Maike Ermster, Jan Gehrmann, Alexander Hapfelmeier, Susann Hueber, Merle Klanke, Christian Kretzschmann, Peter Konstantin Kurotschka, Klaus Linde, Klara Lorenz, Linda Sanftenberg, Antonius Schneider, Stefanie Stark, Til Uebel, Fabian Walter

**Affiliations:** 1 Institute of General Practice and Family Medicine, University Hospital, LMU Munich, Nußbaumstraße 5, 80336 Munich, Germany; 2 Institute of Sociology, Ludwig-Maximilians-Universität, Munich, Germany; 3 Institute of General Practice, Friedrich-Alexander-University of Erlangen-Nuremberg, Erlangen, Germany; 4 Department of General Practice, University Hospital Würzburg, Würzburg, Germany; 5 Institute of General Practice and Health Services Research, Department Clinical Medicine, TUM School of Medicine and Health, Technical University of Munich (TUM), Munich, Germany; 6 General Practice, Faculty of Medicine, University of Augsburg, Augsburg, Germany

**Keywords:** General practice, Primary health care, Participatory research, Practice based research network, Implementation science

## Abstract

**Background:**

Despite general practitioners' (GPs') key role in Germany`s primary health care, clinical research in general practice is scarce. Clinical research is mainly conducted at inpatient facilities, although their results are rarely transferable. German GPs have no extra time or funding for research, as well as limited research training. To support clinical research in German primary health care, practice-based research networks (PBRNs) are developed. As they will be based on an active involvement of GPs, we need more information on GPs` participation-readiness. The aim of this study was to explore facilitators and barriers to participation in the Bavarian Research Practice Network (BayFoNet) from the GPs`perspective before clinical trials will be performed.

**Methods:**

We have performed semi-structured qualitative interviews with a purposive sample of 20 Bavarian GPs in 2022 under the application of the consolidated framework for implementation research (CFIR). Transcriptions were analysed according to Kuckartz` qualitative content analysis. The five domains of the CFIR framework served as initial deductive codes.

**Results:**

*N* = 14 interviewees already agreed to participate in BayFoNet, whereas *n* = 6 interviewees opted not to participate in BayFoNet at the time of data collection. Main facilitators to conduct clinical research within BayFoNet were the motivation to contribute to evidence strength and quality in general practice, professional development and training of practice staff, as well as networking. Barriers for an active participation were bad experiences with previous clinical studies and lack of resources.

**Conclusions:**

PBRNS in Germany have to be promoted and the entire practice team has to be involved at an early stage of development. Professional training of general practice staff and a living network might enhance engagement. Participatory approaches could help to develop acceptable and feasible study designs. Furthermore, PBRNs should support patient recruitment and data collection in general practices and disseminate the results of their research projects regularly to maintain GPs` engagement.

**Trial registration:**

DRKS00028805, NCT05667207.

**Supplementary Information:**

The online version contains supplementary material available at 10.1186/s12875-023-02239-7.

## Introduction

Clinical research in German general practice is scarce, despite the key role of general practitioners (GPs`) in German primary health care. Clinical evidence relevant to decision-making in primary care is predominantly carried out in inpatient settings, although these results are rarely transferable [1–3]. However, there is great potential for clinical research in general practice, where large numbers of patients with a huge variety of risk factors and medical conditions are managed over extended periods of time [4–6]. Consequently, clinical research in general practice has been promoted over the last years in Europe, but research output varies substantially between countries [7, 8]. Whilst some countries (e.g. Canada, the USA, the UK or the Netherlands) have long established practice based research networks (PBRNs), other countries like Germany are progressing more slowly [8–11]. General practices in Germany are independent private businesses, they have no protected time or funding for research. Furthermore, they have typically little research training or experience [12]. Engaging them in teaching or research often depends on intrinsic motivation and a trustful relationship with academic university departments [12, 13]. Consequently, GP practices are ideally integrated into a research infrastructure to bundle competences and conduct relevant, accepted and feasible research of high quality. The German Federal Ministry of Education and Research (BMBF) is therefore funding PBRNs like BayFoNet (Bavarian Research Practice Network). It aims to establish a sustainable PBRN among five regional network centers (RNCs). During the initial five-year funding period, at least two pilot cluster-randomized trials will be conducted. They will provide opportunities to identify faciliators and barriers for a sustainable implementation of BayFoNet [14]. Pilot cluster-randomized trial 1 examines dipsticks and microscopy to reduce antibiotic use in women's uncomplicated urinary tract infections (MicUTI [15]). Pilot cluster-randomized trial 2 investigates the implementation of an online education program for asthma patients in general practice (IMONEDA [16, 17]). MicUTI is registered at Clinical Trials.gov (NCT05667207). IMONEDA is registered at the German Register of Clinical Trials (DRKS00028805). As BayFoNet will be based on an active involvement of GPs, we need more information on GPs` participation-readiness. The aim of this study was to explore facilitators and barriers to participation in the Bavarian Research Practice Network (BayFoNet) from the GPs` perspective before any intervention of these pilot cluster-randomized trials will be performed.

## Material and methods

This study followed the COREQ (COnsolidated criteria for REporting Qualitative research) Checklist [18]. An extended description of methods applied is available in our published study protocol [14].

### Study design

We performed a qualitative study comprising semi-structured interviews with GPs who were actively invited to participate in BayFoNet. The interview guide was based on the „consolidated framework of implementation research (CFIR)“ [19]. Given that general practices consist of individuals operating within an organisation (the practice), which in turn operates within the wider health care system, facilitators and barriers to implement innovations in general practices may be influenced by a multitude of factors at different levels. Therefore, we considered the CFIR a useful framework to systematically explore GPs perspectives on engaging in BayFoNet, which (at its highest level) distinguishes between five types of implementation drivers [19, 20]:

1. Intervention characteristics (for example costs, adaptability, design and packaging of the intervention)
2. Outer setting (external influences on the intervention, such as peer pressure, competitive advantage, patient needs, political measures)
3. Inner setting (internal factors influencing the intervention, such as social architecture or culture within the individual practice)
4. Characteristics of the individual (characteristics of the people affected by the intervention, such as identification with the intervention or attitude towards the topic of an intervention)
5. Process (the process to implement a health care intervention, like planning, organisation, delegation, evaluation).

### Sampling

In a first step, we had invited GPs by information letters to participate in BayFoNet, who had already supported a clinical study at any RNC. Many of those GPs were already part of a teaching network for medical students. Invitations to a research project comprised an additional invitation to participate in BayFoNet as well. To be accredited, GPs and their staff members had to obtain the necessary qualifications in stepwise training courses (module 1–3), whereas a successful completion of module 1 is sufficient for official accreditation. Every training course covers two main ares of interest: a topic of daily patient care in general practice (e.g. deprescribing) and a topic relevant for clinical research in primary care (e.g. patient recruitment in accordance with official ethical guidelines). After official accreditation, GPs received a corresponding certificate as confirmation for their affiliation with BayFoNet (“research practice”). Participation in the primary care-specific training modules could be rewarded with an expense allowance, depending on the qualification level of the training course. Qualified and accredited GPs were invited to participate in one of both pilot cluster-randomized trials. Active participation incorporates different aspects of clinical research, predominantly active patient recruitment as well as project-specific patient education and basic data collection. The concepts of both pilot cluster-randomized trials were developed by GPs, who work in daily patient care and at the same time as research associates at the respective RNC. In a second step, both study designs and interventions were discussed and elaborated in a participatory manner with 10–15 research-interested GPs. Preliminary study material could be requested to facilitate GPs' decision to participate in BayFoNet. Furthermore, we offered project-specific face-to-face trainings for interested practice teams as well as an appropriate compensation for conducting research activities during daily patient care. If the GPs did not answer our invitation letter, we assumed an opt-out to participate actively in BayFoNet at the time of data collection.

To identify facilitators and barriers for participation in BayFoNet from a GPs' perspective, we purposively sampled GPs who were interested in an active participation in BayFoNet, as well as GPs who opted not to participate at the time of data collection. In order to broaden the range of GP perspectives, we also aimed for a mix of male and female GPs, for a mix of rural and urban practices as well as for a mix of solo practices versus group practices.

### Data collection

The interview guide included all five domains of the CFIR and was designed as a problem-centred interview according to Witzel [21]. It was piloted in advance with two physicians in postgraduate training and adapted accordingly (AH, JS).

GPs were interviewed via telephone or video conference according to their own preferences. All respondents were informed verbally about the procedure and objectives of the study before written consent was obtained. No one else was present during these interviews besides the study participant and the researcher. An interview protocol was created for each interview to document formalities (interview code, name of the interviewee, date of the interview, contact details) and special occurrences at the initial contact and during the interview. The interviewer was female medical student (JS), who conducted the qualitative study interviews in a self-reflective, neutral manner [22]. There was no relationship prior to study commencement. At the beginning of each interview, JS introduced herself as a medical student and explained that the results of the interviews will be used for her doctoral thesis. None of the interviews had to be repeated. The transcripts were not returned to the participants for comments or correction. There was no feedback of the participants concerning the results or findings. Interviews were digitally recorded and transcribed verbatim. Interviews were pseudonymized for evaluation. The first interviews were conducted in parallel to the recruitment of interview partners. When further interviews added no additional themes and no further variance within themes, the data was assumed to be saturated.

The interview guide addressed the following topics: general interest and previous experience in clinical research, as well as motivation and individual drivers for/against an active participation in BayFoNet. We wanted to know, if the interviewees plan to take part in one of the upcoming pilot cluster-randomized trials and the main reasons for that decision. Furthermore, we asked for the level of exchange and networking, willingness for personal contributions to make BayFoNet successful, need for support and training to conduct clinical research, as well as long-term wishes and goals in terms of clinical research (Supplementary File 1). Interview partners, who had opted not to participate in BayFoNet and to give an interview, were asked about the main reason for this decision.

### Data analysis

Data were analysed by means of structured content analysis according to Kuckartz [23], whereby deductive and inductive categories were formed. The five domains of the CFIR framework served as initial deductive codes [24]. Text passages were coded supported by MAXQDA22, ordered, further substantiated in terms of content and systematized. By repeatedly reading the entire data set and applying the reduction steps according to Mayring [25], additional inductive codes were formed.

## Results

We interviewed 20 GPs from 20 general practices between March and August 2022. In total, we interviewed *n* = 14 GPs who were active participants in BayFoNet and who did already complete module 1 of the training course at the time of data collection. *N* = 6 GPs who opted not to participate at the time of data collection were also interviewed. Duration of the interviews varied between 3-40min; interviews of GPs who already participated in BayFoNet took 25min. on average.

Most interview partners were male (*n* = 13), worked in a group practice (*n* = 14) in a rural setting (< 10.000 inhabitants; *n* = 16). Half of the interview partners (*n* = 10) planned to participate in an upcoming pilot cluster-randomized trial at the time of data collection (Table 1). None of the interviews had to be repeated or excluded from analysis after data collection.

**Table 1:** Description of the interview partners (*n* = 20 GPs)

	**GPs participating in BayFoNet** ***n* = 14**	**GPs opted out BayFoNet participation** ***n* = 6**
**Female GPs**	*n* = 4	*n* = 3
**Practice located in an urban area (> 100.000 inhabitants)**	*n* = 1	*n* = 3
**Working in a solo practice**	*n* = 1	*n* = 4
**Working in an ambulatory health center**	*n* = 1	*n* = 0
**Number of staff in the practice** **(physicians, physician assistants)**	⌀ = 12	⌀ = 10
**Planned participation in an upcoming pilot cluster-randomized trial of BayFoNet (MicUTI/ IMONEDA) **	*n* = 10	*n* = 0

The following presentation of the results is based on the interview guide (see Supplementary File 1). "(…)" means a break in the narrative flow, "[…]" means a shortening of the quote.

Four consistent themes merged from our analysis of GP interviews:

1. Evidence strength and quality in general practice

Most GPs mentioned the need for evidence strength and quality in general practice as major facilitator for their motivation to support BayFoNet. Study design should be relevant and feasible for patient care in daily practice. On a long term, this aspect might help to strengthen the voice of general practice in health policy.

2) Professional development and training of practice staff

Regular primary-care specific traning sessions were pronounced drivers for GPs` willingness to be part of BayFoNet and to conduct clinical trials. Vice versa, an overwhelming study design without any research training and well-integrated processes were identified as possible barriers.

3) Networking with other GPs and academic general practice

To collaborate with colleagues and academic general practice was very important for our study particpants. However, being part of BayFoNet was not perceived as a competitive advantage. Many interviewees, who opted not to participate in BayFoNet, did not perceive any personal connection or participatory exchange with academic general practice so far.

4) Available resources

Most study participants emphasized that a financial incentive was not the main facilitator. A reliable contact person at the university department in addition to a traveling study nurse would be very helpful. Consequently, a lack of staffing in one's own practice was mentioned by most interviewees as a main barrier.

1. Evidence strength and quality in general practice

When we asked for the main drivers to participate actively in BayFoNet, providing evidence strength and quality for the treatment of patients in general practice was pronounced by all interviewees, who already decided to participate in BayFoNet.

*"If the study gives insights and perspectives for further treatment. I think it is worth it. We need a theoretical backup. I can't treat my patients just based on assumptions or feelings or experiences, we need clear evidence to treat patients.“ (Interview Transcript OT, Pos. 39).*

"*Much of what is written and done in medical research is important. But that has nothing to do with daily practice. For this reason, research in general practice is important. I've thought for a long time that it would actually be very important to collect and analyze data from daily practice. And that's why I think it's great that it's now possible to participate in BayFoNet.” (Interview Transcript IX, Pos. 33).*

When we asked for the main reasons for participation in a pilot cluster randomised trial within BayFoNet, our interview partners were talking mainly about the study designs of both pilot cluster randomised trials as well as the outer appearance or handling of the preliminary study material. The optimal study design should be easy to integrate into everyday practice.

*“So far I am very satisfied (…) that the study is already prepared. For example, that all documents are pre-designed, that return envelopes are included, and that everything is somehow simple." (Interview Transcript IX, Pos. 21).*

Study design and the research questions have to be relevant and feasible for GPs, their staff and the patients in general practice. Considering their individual motivations and abilities, an active involvement of patients in clinical research could increase their appreciation.

*“And the opportunity to offer my patients an added value in daily practice. […] That is also something pleasant if the collaboration is interesting for the patients. […] It's appreciated, if someone has a medical issue and can speak up, that's always nice. Even if it's just a questionnaire. I think that's a good thing.“ (Interview Transcript FE, Pos. 35).*

For some interviewees, evidence strength and quality in general practice is not only important to inform clinical decision making and improve patient care, but also helpful to strengthen the voice of general practice in health policy. This is an important vision for some interviewpartners for the upcoming years.

*“What we are doing right now, the ability to speak and argue, also in a political context. I hope that we will get there with BayFoNet in five years. “ (Interview Transcript JR* *,* *Pos. 39)*

2) Professional development and training of practice staff

Another important aspect that motivated our study participants to become part of BayFoNet, was a regular professional development and training of practice staff.

“*It would be a great success if BayFoNet could make easy-to-understand scientific training possible for general practitioners and their employees. Because I always have to keep myself up to date. The second point is to contribute to scientific work, which also educates you.“ (Interview Transcript FE, Pos. 35).*

*“We really like the opportunity to continue our professional education. Your colleagues have done quite well with the video tutorials that can be called up online. Where all these study types and techniques have been explained. I have heard it all before. What kind of studies are there? How must they be carried out? Therefore, that was good and it makes sense in general practice area and is easy to implement during daily business.“ (Interview Transcript VG, Pos. 31).*

On the other hand, when GPs start to conduct clinical research in daily practice, it is of utmost importance to give as much support as possible. Every person involved in the study performance has to be trained, all roles and responsibilities should be clearified in advance. Bad experiences with previous clinical trials can permanently destroy the motivation for further support of clinical research.

*"We participated in a study on the subject of Covid-19 and that was totally difficult and tough. Because the employees and colleagues could not integrate it in their routines. You had to recruit people and it was tough to get the minimum number of recruited patients. I would say from that single experience, I don't do that anymore." (Interview Transcript LE, Pos. 7).*

3) Networking with other GPs

When we asked for external strategies to spread BayFoNet including policy, collaborations and public or benchmark reporting, most of the study participants perceived collaboration with other GPs and networking with universities as important incentives. This was particularly relevant for practices in rural areas.

*“A university connection and networking with other colleagues is always exciting and would certainly be a great incentive for many lone fighters, but here in the countryside it is really difficult.” (Interview Transcript NS, Pos. 45).*

At the time of the survey, many interviewees missed such networking. However, the interviewees do not expect a competitive advantage through participation in BayFoNet. Since they work primarily in underserved areas, the external impact of the BayFoNet certificate (”research practice”) was not considered to be high. Although the practices were run as private companies, additional advertising media to attract more patients or an unique selling point compared to other general practices was apparently not needed.

*"I'd be happy if I would need a competitive advantage! We are an underserved area here. Send in some competitors that would be good. I wouldn't mind." (Interview Transcript NK* *,* *Pos. 67)*

*"I don't think that participating in BayFoNet is real advantage in comparison to other general practices, to be honest. Because then you would have to actively post it somewhere and advertise it. (…)In general practice, a real personal doctor-patient relationship is important. (…) Even if the other GPs are teaching students or whatever. I can't imagine that people will talk about it.” (Interview Transcript IX, Pos. 35).*

Many interviewees who opted out to participate in BayFoNet at the time of data collection did not perceive any personal connection to academic general practice. A lively network was not expected, a participatory exchange to develop relevant and feasible research questions and study designs had not taken place so far.

*“Of course, a connection to university is always exciting, but in reality, how strong is the connection? Well, it's not like you get together once a month and talk about research results, and I don't know how it is in your network, to what extent you really bring people together and network them, but I don’t expect much here.” (Interview Transcript NS, Pos. 45).*

*“But this is a kind of data collection for the institute. Apart from supporting clinical research, I don't see any great benefit from that."(Interview Transcript NS* *,* *Pos. 39)*

4) Available resources

Available resources determine the feasibility of clinical studies within the inner setting of general practices. Financial incentives were not of great importance among our study participants. However, it is understandable that GPs do not want to pay for any necessary resources themselves.

* “I don't really care about the finances myself. […] For me it would be more important that the institute provides the necessary resources to get things done, than paying me money to provide the resources.” (Interview Transcript NS, Pos. 19).*

Accordingly, traveling study nurses who can be deployed flexibly were seen as important facilitators for practices’ engagement in research.

“*And as I said, the most important thing is that these are all studies that can be conducted in terms of time. Or that there is a study nurse available for participating practices to support them.” (Interview Transcript TB, Pos. 47).*

When we asked for currently available tools and support that are already used by the study participants, they refered mainly to a reliable contact person at the university.

*"I am very happy with the support that's been given. That you have a contact person that is always easy to reach.” (Interview Transcript IX* *,* *Pos. 21)*

Consequently, missing opportunities to delegate tasks and responsibilities due to a lack of resources and staff was the most important perceived barrier for our interviewees to engage themselves and their staff in clincal research.

*"But for me it is actually a resource problem. I cannot delegate someone to fill out a questionnaire for each patient or anything. We often have inquiries about that.“ (Interview Transcript NS, Pos. 21).*

*“In contrast to the hospital - there you have trained staff or study nurses who do that. In practice, a lot depends on the nurses. It is really busy in general practice! Time is the most precious factor. Moreover, if you have something to add to a study, nobody likes doing that. And if it's very extensive additional work, well….“ (Interview Transcript AF, Pos. 27).*

## Discussion

### Statement of principal findings

Providing evidence and quality to improve patient care were main facilitators for network attendance, as well as professional development and training of practice staff. Another aspect of participation-readiness in BayFoNet was interest in networking with colleagues and academic departments of general practice. Most interviewed GPs were not interested in primary financial incentives. Participatory approaches might help to overcome main barriers to GPs engagement in clinical research, as time-saving and resource efficient study designs, as well as a need for reliable support during the performance of clinical trials was emphasized.

### Strengths and weaknesses of the study

This study provides important insights into GPs expectations regarding an active PBRN participation in Bavaria. These findings could be helpful in the upcoming development of BayFoNet in the next years, as well as PBRN development in other health care systems. PBRNs could be geared to the needs of the target group, while also optimizing upcoming research project according to relevance, acceptance, credibility and feasibility. The study-involvement of GPs from different areas, setting and levels of commitment concerning clinical research increase the explanatory power and support generalizability of our findings.

However, the majority of the interviewees were male, and a more balanced study sample would have been desirable. We only addressed GPs in this study and not the practice staff, despite their central role in patient recruitment for medical studies. Their needs should be subject to further research, as well as the patients` perspective. Furthermore, we only assessed the rudimentary expectations and voiced intentions of research interest at a very early development phase of BayFoNet in this analysis. To what extent this interest and expectations will be translated into real research activity is yet to be assessed.

### Findings in relation to other studies

German research output in general practice remains unsatisfactory, which is at least partly attributable to deficits in research infrastructure [26]. As German GPs work in a market-based, competitive setting of small private practices, they have no protected time or funding for research. As these characteristics of the German primary care practice setting are comparable to other PBRN practices, it should be feasible to overcome at least some of the identified barriers [8]. Academic departments of general practice depend mainly on scarce public research funding [12]. Improving evidence and the quality of patient care were identified as main motivators in focus groups and surveys of primary care clinicians of international PBRNs as well [27, 28]. Immediate or impending benefits to their clinical practice and patient population would be a great facilitator for network attendance, despite potential work constraints.

To improve research performance in general practice, knowledge acquisition is fundamental [27, 29]. German GPs have typically no or little research training or experience, whereas Good Clinical Practice (GCP) training is often compulsory for participation in clinical trials [12]. Consequently, our study participants’ referred very often to the importance of professional development and training courses offered via BayFoNet. In accordance with our results, German GPs of other PBRNs favour compact, remote training moduls covering research-relevant topics, which keep practices up-to-date and foster learning and exchange within the network [30].

Interviewees, who plan to be active BayFoNet participants, were mainly GPs, who work in group practices in a rural setting. Consequently, another important facilitator for participation-readiness was networking with other GP practices and academic departments of general practice. Positive experiences in previous cooperation as well as the reputation of an academic department have already been identified as important drivers for GPs` motivation to be part of a German PBRN [31]. Furthermore, a mutual basis of trust and a reliable contact person are known prerequisites for a strong relationship between general practices and academic institutions [29]. Cosmopolitanism is indicated by the degree to which a person or organization networks externally on an informal interpersonal or interorganizational basis [32]. Innovations and changes in daily practice are implemented more likely by organizations that support and promote external boundary-spanning roles [32, 33]. Therefore, cosmopolitanism, interest in networking and communication might be local cultural indicators of suitable participating practices in PBRNs. GPs who agreed to participate in BayFoNet considered a certified affiliation and the associated external impact with a university as a less important driver for network participation, than GPs who opted out network cooperation so far. This might be due to already existing teaching activities and corresponding affiliation with a university (“teaching practice”) of most interested GPs [34]. To enable successful, resource-efficient and sustainable clinical research in German primary care, a long-term cooperative connection between GPs and academic departments has to be created [31, 34].

Good communication throughout all phases of medical research with the GPs and the involved staff is particularly important to facilitate and improve practice-based research and develop relevant, accepted and feasible clinical studies [28, 30]. Although financial incentives are not primary drivers for participation, an adequate remuneration of time investments is provided within BayFoNet, and ensures that lack of remuneration is not a barrier for active participation. In order to enable continuity and sustainability in a research network, Australian GPs would appreciate a reimbursement for additional costs as well [35]. Resource efficient study designs and the delegability of individual tasks are very important for GPs, which operate as private businesses in Germany [36]. Therefore, the need to invest time during consultation hours is a major barrier for GPs’ participation in medical research [37, 38]. In general, attractiveness and credibility of medical research will be positively affected by a low-time effort for GPs and their staff [39]. PBRNS in Germany have to be promoted and the entire practice team has to be involved at an early stage of development. Research processes should be adapted individually as far as possible to the respective practice and patient recruitment should be avoided during consultation hours [40]. Data collection and follow-up by dedicated traveling nurses could reduce the administrative burden and improve the recruitment of both practitioners and patients [41, 42].

### Meaning of the study

We found interest in conducting clinical research projects, whereas the benefit of an active participation in BayFoNet has to be elaborated and communicated more precisely. I would be helpful to share a mission and/or vision statement clearly at the time of practice recruitment, which then would help to convey a clear understanding of the purpose of membership. This may refer to the anticipation of an increased workload, time investment and the German GPs` inexperience in PBRNs. Furthermore, we have decided to invite GPs for participation in BayFoNet, independent of recruitment for a clinical trial, but based on regular remote professional training for general practice teams in 2023 and 2024. The relevance and interest in the respective research topic for daily clinical practice is one of the main reasons for GPs involvement [41]. To define research topics in a top-down approach while simultaneously facilitating a bottom-up selection process is important to identify topics relevant to GPs and their patients [30]. Within the European general practice research agenda 2010, medical research on common complaints in non-selected patients have been already defined as a top priority (1). This resolution emphasizes the high importance of research on everyday issues in the real life setting of primary care and connecting German PBRNs and the international primary research community.

## Conclusion

It is highly important to elaborate and communicate benefits for GPs participation in clinical research and PBRNs. Future efforts should promote and prioritize practice-driven research topics and enable GP teams to perform them. A low and predictable time effort should be ascertained in clinical studies, as well as a reliable contact person at university. Our findings are relevant for the development of primary care research and PBRNs in general practice settings on a national level and may guide recruiting strategies and constituting networks in other countries as well.

### Supplementary Information

**Additional file 1.** Interview guide for GPs.

## Data Availability

Data and materials might be obtained from the authors upon reasonable request.

## Abbreviations

BayFoNet: Bavarian Research Practice Network
CFIR: Consolidated framework for implementation
GP: General practitioner
PBRN: Practice-based research network
RNC: Regional network centre
