# Supplementary material for: Expectations of general practitioners on a practice based research network in Germany- a qualitative study within the Bavarian Research Practice Network (BayFoNet)
Source: BMC Prim Care. 2024 Jan 2;25:10. doi: 10.1186/s12875-023-02239-7 (PMC10759500; doi:10.1186/s12875-023-02239-7)
Supplement: Supplementary file 1 — Additional file 1. Interview guide for GPs. [file 12875_2023_2239_MOESM1_ESM.docx]

| **time point** | **before intervention** |
| --- | --- |
| **CFIR domain** | **research questions** |
| intervention characteristics | What would make "BayFoNet" successful for you? |
|  | What tools and support are currently available and how do you use them? |
| outer setting | What can others do to make "BayFoNet" successful? |
| inner setting | Do you know of any other practice-based research networks? |
|  | Do you know other "BayFoNet" practices? |
| characteristics of the individual | Why do you participate in "BayFoNet"? |
|  | What can you contribute to make "BayFoNet" successful? |
|  | What are your hopes and wishes for "BayFoNet"? |
|  | What previous research experience do you have? |
|  | What kind of support do you need? |
| process | How was "BayFoNet" introduced to your practice team? |

Supplementary file 1: Interview guide for GPs
